# miR-424(322) reverses chemoresistance via T-cell immune response activation by blocking the PD-L1 immune checkpoint

**DOI:** 10.1038/ncomms11406

**Published:** 2016-05-05

**Authors:** Shaohua Xu, Zhen Tao, Bo Hai, Huagen Liang, Ying Shi, Tao Wang, Wen Song, Yong Chen, Jun OuYang, Jinhong Chen, Fanfei Kong, Yishan Dong, Shi-Wen Jiang, Weiyong Li, Ping Wang, Zhiyong Yuan, Xiaoping Wan, Chenguang Wang, Wencheng Li, Xiaoping Zhang, Ke Chen

**Affiliations:** 1 Department of Gynecology, Shanghai First Maternity and Infant Hospital, Tongji University School of Medicine, Shanghai 201204, China; 2 Department of Radiation Oncology, Key Laboratory of Cancer Prevention and Therapy, Tianjin Medical University Cancer institute & Hospital, National Clinical Research Center of Cancer, Tianjin 300060, China; 3 Department of Urology, Union Hospital, Tongji Medical College, Huazhong University of Science and Technology, Wuhan 430022, Hubei, China; 4 Department of Urology, Tongji Hospital, Tongji Medical College, Huazhong University of Science and Technology, Wuhan 430030, Hubei, China; 5 Emergency Center, Tongji Hospital, Tongji Medical College, Huazhong University of Science and Technology, Wuhan 430030, Hubei, China; 6 Department of Gynecology, Changzhou Maternal and Child Health Hospital Affiliated to Nanjing Medical University, Changzhou 213003, China; 7 Department of Biomedical Science, Mercer University School of Medicine, Savannah, Georgia 31404, USA; 8 Department of Pharmacy, Union Hospital, Tongji Medical College, Huazhong University of Science and Technology, Wuhan 430022, Hubei, China; 9 Key Laboratory of Tianjin Radiation and Molecular Nuclear Medicine, Institute of Radiation Medicine, Peking Union Medical College & Chinese Academy of Medical Sciences, Tianjin 300308, China

## Abstract

Immune checkpoint blockade of the inhibitory immune receptors PD-L1, PD-1 and CTLA-4 has emerged as a successful treatment strategy for several advanced cancers. Here we demonstrate that miR-424(322) regulates the PD-L1/PD-1 and CD80/CTLA-4 pathways in chemoresistant ovarian cancer. miR-424(322) is inversely correlated with PD-L1, PD-1, CD80 and CTLA-4 expression. High levels of miR-424(322) in the tumours are positively correlated with the progression-free survival of ovarian cancer patients. Mechanistic investigations demonstrated that miR-424(322) inhibited PD-L1 and CD80 expression through direct binding to the 3′-untranslated region. Restoration of miR-424(322) expression reverses chemoresistance, which is accompanied by blockage of the PD-L1 immune checkpoint. The synergistic effect of chemotherapy and immunotherapy is associated with the proliferation of functional cytotoxic CD8+ T cells and the inhibition of myeloid-derived suppressive cells and regulatory T cells. Collectively, our data suggest a biological and functional interaction between PD-L1 and chemoresistance through the microRNA regulatory cascade.

## Abstract (web summary)

Resistance to chemotherapy occurs in many ovarian cancer cases. Here, the authors show that mir-424(322) expression restores the sensitivity of ovarian cancer cells to chemotherapy by blocking the PD-L1 immune checkpoint, and find that combining immunotherapy and chemotherapy has a synergistic effect.

## Graphical abstract

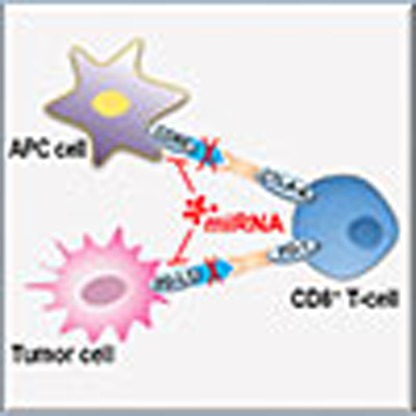

Epithelial ovarian carcinoma is the leading cause of death in women with gynaecological malignancies^1^. Despite the current multidisciplinary treatments, the overall prognosis remains poor^2^,^3^. More than 75% of patients diagnosed with epithelial ovarian carcinomas are at an advanced stage of the disease, and the 5-year survival rate is <30% (ref. 4). Chemotherapy is one of the most effective and commonly used treatments for ovarian cancer. However, the development of chemoresistance limits its clinical application in ovarian cancer patients^5^.

The immune system affects developing cancers by functioning as an extrinsic tumour suppressor that destroys developing tumours or restrains tumour expansion^6^. However, tumour cells could escape surveillance by immune cells. Tumour immune evasion is considered an important hallmark of cancer initiation and progression^7^. Programmed death-1 (PD-1) and T-lymphocyte associated antigen-4 (CTLA-4) are immunomodulatory receptors expressed in T-cell membranes^8^,^9^. Programmed death-1 ligand 1 (PD-L1), which binds to the PD-1 receptor, is expressed in tumour and/or macrophage cells, whereas CD80, which binds to the CTLA-4 receptor, is expressed in dendritic cells (DCs)^10^. The PD-L1/PD-1 and CD80/CTLA-4 interactions inhibit CD8+ cytotoxic T-lymphocyte proliferation and survival and affect the function of tumour-infiltrating T cells, which suppress the immune system and cause peripheral immune tolerance in cancer patients^11^. Immune checkpoint blockade of the inhibitory immune receptors PD-1 and CTLA-4 and immune ligand PD-L1 has emerged as a promising treatment strategy for several advanced cancers^11^. Recent studies have demonstrated an upregulation of PD-L1 expression in cancer cells by chemo-preventive agents and a resulting decrease in cancer cell-specific T-cell activity promoted immune evasion^12^. These findings suggest a potential link between chemotherapy and immunoresistance in ovarian cancer.

MicroRNAs (miRNAs) are a class of small noncoding RNA molecules that post-transcriptionally modulate gene expression by binding to the 3′-untranslated region (3′-UTR) of target genes^13^. Individual miRNAs often target multiple transcripts rather than one specific gene and one mRNA could be targeted by a group of miRNAs. The majority of studies on miRNAs have focused on their function as an oncogene or tumour suppressor. Recently, accumulating evidence has indicated that miRNAs play important roles in the regulation of the host immune response^14^. miR-34a, miR-200 and miR-513 have been demonstrated to translationally regulate PD-L1 expression^12^,^15^,^16^,^17^. However, whether miRNAs are directly involved in the transcriptional regulation of PD-L1/PD-1 and/or CD80/CTLA-4 immune expression remains unclear.

In this study, we analysed the 3′-UTR of the PD-L1, PD-1, CD80 and CTLA-4 genes and demonstrated that both PD-L1 and CD80 are potential targets of the miR-15 family, which includes miR-15a, miR-15b, miR-16, miR-195, miR-424, miR-497 and miR-503. However, only miR-424(322) was inversely correlated with PD-L1, PD-1, CD80 and CTLA-4 expression in a clinical gene-expression array data set. High expression levels of miR-424(322) were positively correlated with the PFS of ovarian cancer patients. miR-424(322) overexpression reduced PD-L1 and CD80 expression through direct binding to the 3′-UTR of these genes. Furthermore, low miR-424(322) and high PD-L1 expression were significantly correlated and strongly associated with chemoresistant phenotypes in ovarian cancer cells and tissues. Restoration of miR-424(322) expression enhanced the sensitivity of cancer cells to drug treatment and was accompanied by T-cell activation by blocking the PD-L1 immune checkpoint in both *in vitro* and *in vivo* models. Our current findings indicate that miR-424(322) regulates PD-L1 and CD80 expression. Therefore, miR-424(322) might serve as a therapeutic target to enhance the chemosensitivity of ovarian cancer cells through checkpoint blockage, which thereby promotes the T-cell response in attacking tumour cells.

## Results

### miR-424(322) expression is inversely correlated with PD-L1

**Figure 1:**
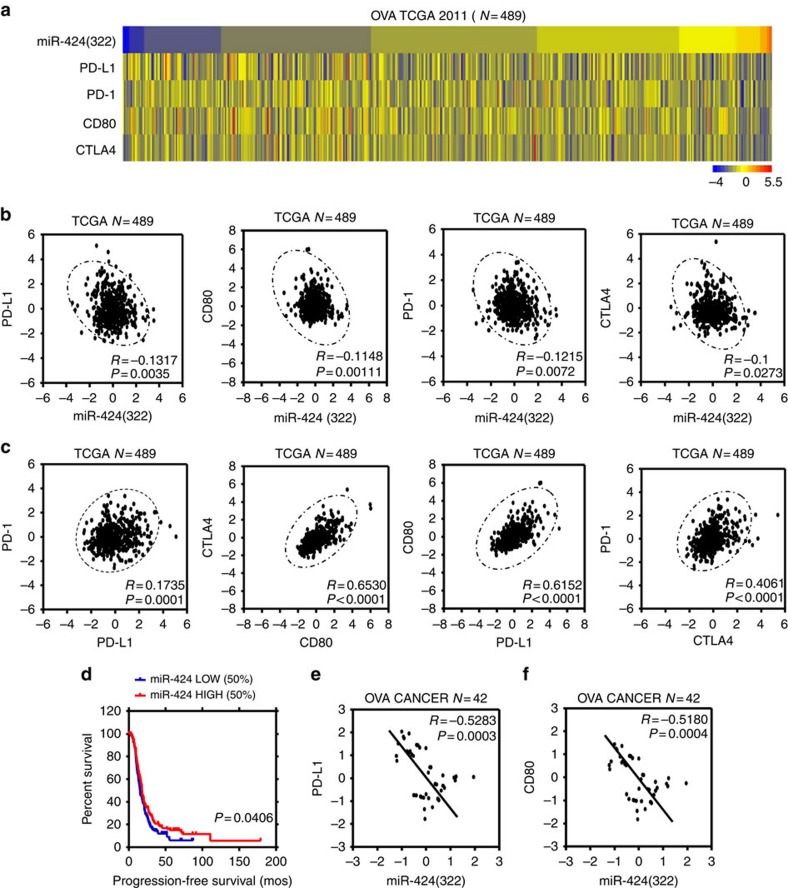
miR-424(322) is in versely correlated with PD-L1 levels in ovarian cancer patients. (**a**) Heat map depicting samples from TCGA 2011 combined human ovarian cancer microarray data sets that were assigned to ovarian cancer gene expression subtypes (*n*=489). (**b**) miR-424(322) levels were inversely correlated with PD-L1 (*r*=−0.1317, *t-test*, *P*=0.0035), PD-1 (*r*=−0.1215, *t-test*, *P*=0.0072), CD80 (*r*=−0.1148, *t-test*, *P*=0.0011) and CTLA-4 (*r*=−0.1, *t-test*, *P*=0.0273) expression levels in human ovarian cancer. (**c**) PD-L1 expression levels were positively correlated with PD-1 expression levels in human ovarian cancer (*r*=0.1735, *t-test*, *P*=0.0001); CD80 expression levels were positively correlated with CTLA-4 expression levels in human ovarian cancer (*r*=0.6530, *t-test*, *P*<0.0001); PD-L1 expression levels were positively correlated with CD80 expression levels in human ovarian cancer (*r*=0.6152, *t-test*, *P*<0.0001); CTLA-4 expression levels were positively correlated with PD-1 expression levels in human ovarian cancer (*r*=0.6530, *t-test*, *P*<0.0001). (**d**) Kaplan–Meier analysis for miR-424(322) indicates that the patients with samples in which miR-424(322) was highly expressed (50% high), have improved progression-free survival (*t-test*, *P*=0.0406). (**e**,**f**) Spearman's rank correlation analysis identified inverse correlations between miR-424(322) and PD-L1 (*r*=−0.5283, *t-test*, *P*=0.0003) and CD80 (*r*=−0.5180, *t-test*, *P*=0.0004) in the ovarian cancer tumour samples.

miRNAs post-transcriptionally modulate gene expression by binding to the 3′-UTR of target genes. Recent studies demonstrated that miRNAs play important roles in the regulation of the host immune response^14^. We first analysed the 3′-UTR sequences of PD-L1, PD-1, CD80 and CTLA-4 genes using the public database *microRNA.org* and determined that the PD-L1 and CD80 immune checkpoint genes are potential targets of the miR-15a/15b/16/195/424/497/503 family (Supplementary Fig. 1A,B). To further evaluate the physiological relevance of PD-L1/PD-1/CD80/CTLA-4 and miR-15a/15b/16/195/424/497/503 family interactions, we performed *in silico* analyses of miR-15a/15b/16/195/424/497/503 family members and PD-L1/PD-1/CD80/CTLA-4 expression using a 2011 TCGA data set composed of 489 ovarian cancer patient samples. As shown in Fig. 1a–c and Supplementary Fig. 2A–H, only miR-424(322) was inversely correlated with PD-L1, PD-1, CD80 and CTLA-4 expression, whereas a positive correlation was identified among PD-L1, PD-1, CD80 and CTLA-4 expression. Furthermore, a Kaplan–Meier analysis was conducted to determine whether the disease PFS of patients was associated with expression of miR-15a/15b/16/195/424/497/503 family members in tumours. miRNA expression was used to assign patients to the high (upper 50th percentile) or low (lower 50th percentile) expression groups. A Kaplan–Meier analysis indicated that the patients with tumours that expressed high levels of miR-424(322) had a longer disease PFS (*P*=0.04) (Fig. 1d, Supplementary Fig. 3A–F).

To further confirm the interaction between miR-424(322) and PD-L1/CD80, we examined the miR-424(322) and PD-L1/CD80 levels with quantitative real-time PCR (qRT-PCR) in ovarian cancer samples. As shown in Fig. 1e,f, miR-424(322) was inversely correlated with PD-L1 and CD80. Taken together, these findings suggest that miR-424(322) modulates the immune response by interacting with the PD-L1/PD-1 and CD80/CTLA-4 immune checkpoint genes in ovarian cancer.

### PD-L1 and CD80 are directly regulated by miR-424(322)

**Figure 2:**
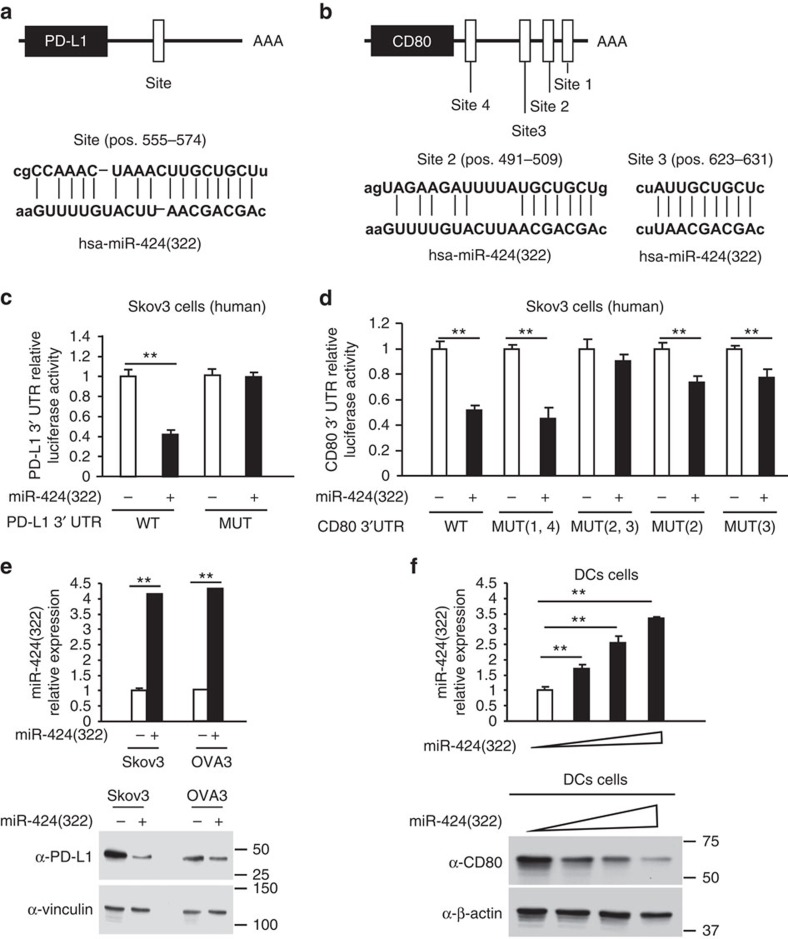
PD-L1 and CD80 are directly regulated by miR-424(322). (**a**,**b**) PD-L1 and CD80 are potential targets of miR-424(322). The miR-424(322)-targeting sites in the 3′-UTRs of human PD-L1 and CD80 are shown. (**c**,**d**) The luciferase vectors that contain the human wild-type (WT) and mutant (MUT) PD-L1 (**c**) and CD80 (**d**) 3′-UTR regions were co-transfected into Skov3 cells with miR-424(322) or scramble miRNA precursors (miR-Scr). The relative luciferase/*Renilla* activities were analysed in the cells 48 h after the transfection. The results represent the mean±s.e.m. from three independent experiments. *t-test*, **P≤0.01. (**e**) Skov3 and OVA3 cells were transfected with miR-424(322) or miR-Scr. mir-424(322) mRNA levels were determined via qRT-PCR assay. *t-test*, **P≤0.01. The results represent the mean±s.e.m. from three independent experiments. The expression levels of PD-L1 were analysed by western blotting. One representative experiment of three experiments is shown. (**f**) DCs were transfected with miR-424(322) or miR-Scr. miR-424(322) mRNA levels were determined via a qRT-PCR assay. *t-test*, **P≤0.01. The results represent the mean±s.e.m. from three independent experiments. The expression levels of CD80 were determined by western blotting. One representative experiment of three experiments is shown.

To validate the bioinformatics analysis, which indicated that PD-L1 and CD80 were potential targets of miR-424(322), luciferase reporter assays were performed with wild-type PD-L1 3′-UTR, wild-type CD80 3′-UTR and mutants with deletions of the putative miR-424(322) binding sequences (Fig. 2a,b, Supplementary Fig. 4A). As shown in Fig. 2c, the overexpression of miR-424(322) inhibited wild-type but not mutant luciferase reporter activity in Skov3 cells, suggesting that miR-424(322) might target PD-L1 through the 3′-UTR binding site. Similarly, as shown in Fig. 2d, miR-424(322) overexpression significantly inhibited activity from the wild-type CD80 3′-UTR and CD80 3′-UTR mutated at sites 1 and 4 and slightly inhibited CD80 3′-UTR mutated at sites 2 and 3, suggesting that miR-424(322) might target CD80 through the 3′-UTR sites 2 and 3. Western blot analyses subsequently demonstrated that miR-424(322) overexpression in Skov3 and OVA3 human cancer cells decreased the protein levels of PD-L1 (Fig. 2e), and miR-424(322) overexpression in human DCs decreased the protein levels of CD80 (Fig. 2f). Taken together, these findings indicate that miR-424(322)-mediated suppressions of PD-L1 and CD80 expression requires the 3′-UTR of the PD-L1 and CD80 genes.

### miR-424(322) regulates cytokine secretion by blocking PD-L1

**Figure 3:**
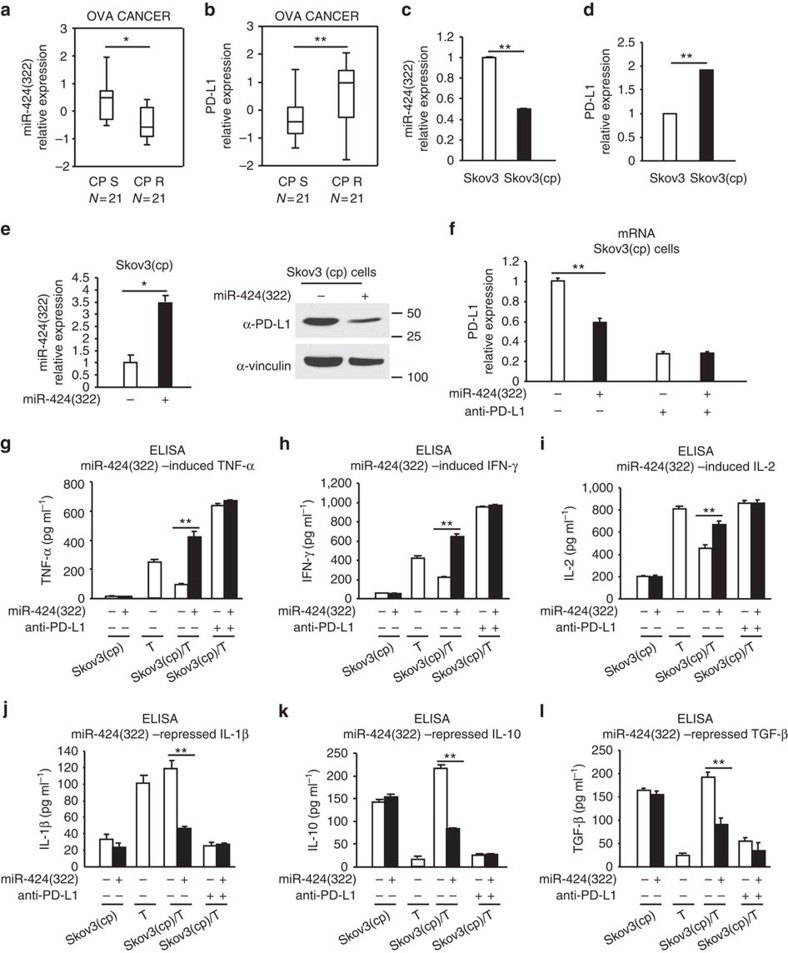
miR-424(322) regulates T-cell cytokine secretions by blocking PD-L1 in a Skov3 (CP)/T-cell co-culture model. (**a**,**b**) RT-PCR was performed to determine miR-424(322) and PD-L1 expression in 21 platinum-sensitive and 21 platinum-resistant ovarian tumours. (**a**) miR-424(322) levels were significantly decreased (*t-test*, **P≤0.01) and (**b**) PD-L1 levels were significantly increased (*t-test*, **P≤0.01) in the platinum-resistant tumours compared with the platinum-sensitive tumours. (**c**,**d**) RT-PCR was performed to determine miR-424(322) and PD-L1 expression in Skov3 and Skov3 (CP) cells, respectively. (**c**) miR-424(322) levels were significantly decreased (*t-test*, **P≤0.01) and (**d**) PD-L1 levels were significantly increased (*t-test*, **P≤0.01) in the Skov3 (CP) cells compared with the Skov3 cells. The results represent the mean±s.e.m. from three independent experiments. (**e**) Skov3 (CP) cells with stable overexpression of miR-424(322). miR-424(322) mRNA levels were determined via qRT-PCR assay. *t-test*, *P≤0.05. The results represent the mean±s.e.m. from three independent experiments. PD-L1 protein levels were determined by western blotting. One representative experiment of three experiments is shown. (**f**–**l**) Skov3 (CP) cells with stable overexpression of miR-424(322) with or without PD-L1 blocking antibody (anti-PD-L1). After 24 h, T cells were subsequently co-cultured with mitomycin C-treated Skov3 (CP) cells for 24 h. (**f**) Skov3 (CP) cells were sorted by FACS. The relative expression levels of PD-L1 in the Skov3 (CP) cells were determined via qRT-PCR assay. *t-test*, **P≤0.01. The results represent the mean±s.e.m. from three independent experiments. (**g**–**l**) Co-culture media were assayed for TNF-α, IFN-γ, IL-2, IL-10, IL-1β and TGF-β by cytokine ELISA assay. *t-test*, **P≤0.01. The results represent the mean±s.e.m. from three independent experiments.

The upregulation of PD-L1 expression in cancer cells by chemotherapeutic agents and the resulting decrease in cancer cell-specific T-cell activity might promote immune evasion of tumour cells^12^, which suggests a potential link between chemotherapy and immunoresistance. To determine whether miR-424(322) and PD-L1 are involved in ovarian cancer chemoresistance, we investigated the association between the miR-424(322) and PD-L1 levels in primary ovarian tumours and their response to platinum-based chemotherapy. The miR-424(322) levels were significantly decreased and the PD-L1 levels were significantly increased in the platinum-resistant tumours compared with the platinum-sensitive tumours (Fig. 3a,b). We subsequently examined the expression of miR-424(322) and PD-L1 in Skov3 and Skov3 (CP) (Skov3 cisplatin resistant) cells. miR-424(322) was significantly decreased and PD-L1 was significantly increased in the Skov3 (CP) compared with the Skov3 cells (Fig. 3c,d). Furthermore, the restoration of miR-424(322) expression in Skov3 (CP) cells resulted in decreased PD-L1 expression (Fig. 3e), suggesting that miR-424(322) plays an important role in immune evasion in chemoresistant ovarian cancer.

The release of cytokines is critical for many aspects of T-cell function^18^. The PD-L1/PD-1 immune checkpoint pathway has been demonstrated to inhibit the T-cell anti-tumour immune response^11^, and PD-L1 preferentially modulates the secretion of regulator cytokines in T cells^19^. We assessed if increased PD-L1 expression in chemoresistant ovarian cancer cells affects tumour-reactive T-cell function. We also examined whether miR-424(322) is involved in this process. We established a Skov3 (CP)/T-cell co-culture model. Co-culturing T cells with miR-424(322) mimics transfected Skov3 (CP) cells increased TNF-α, IFN-γ and IL-2 and decreased IL-1β, IL-10 and TGF-β secretion in the media. The effect of anti-PD-L1 was similar to the effect of miR-424(322) (Fig. 3f–l), suggesting that miR-424(322) modulates cytokine secretion in T cells by blocking a PD-L1/PD-1-dependent pathway. Taken together, these findings demonstrate that the suppression of PD-L1 expression by miR-424(322) might affect tumour-reactive T-cell functions in chemoresistant ovarian cancer.

### miR-424(322) influences PD-L1-associated T-cell apoptosis

**Figure 4:**
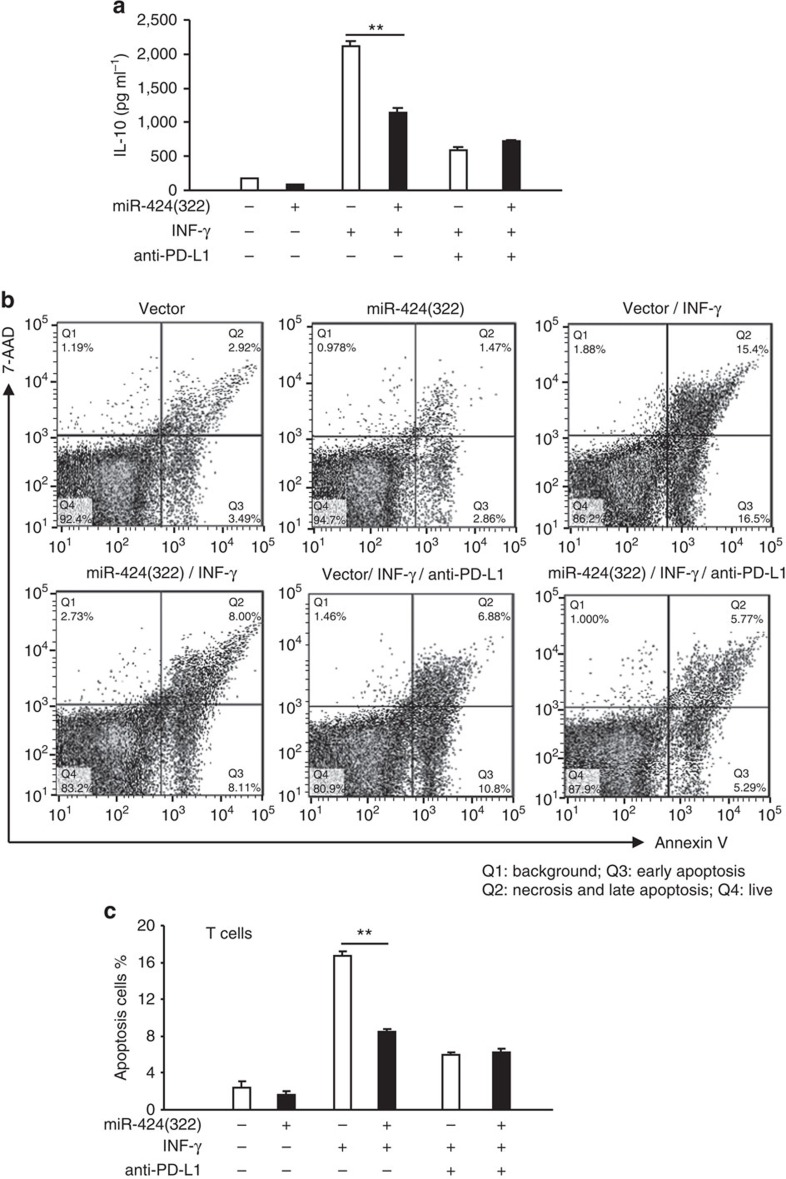
miR-424(322) influences IFN-γ induced PD-L1-associated CD8+ T-cell apoptosis in a Skov3 (CP)/T-cell co-culture model. Skov3 (CP) cells with stable overexpression of miR-424(322) or miR-Src were first exposed to IFN-γ for 24 h in the presence or absence of anti-PD-L1. (**a**) Culture media were assayed for IL-10 using a cytokine ELISA assay. *t-test*, **P≤0.01. The results represent the mean±s.e.m. from three independent experiments. (**b**,**c**) T cells were subsequently co-cultured with mitomycin C-treated Skov3 (CP) cells for 24 h. T cells were sorted by FACS, stained with PE anti-PD-L1, Alexa Fluor 488 anti-annexin V and APC anti-CD8, and analysed by flow cytometry for T-cell apoptosis in the PD-L1+/CD8+ population. *t-test*, **P≤0.01. The results represent the mean±s.e.m. from three independent experiments, and the densitometric level of the apoptosis ratio is shown.

T cells are the key mediators of tumour surveillance and contribute to the adaptive immune response^20^. Following the recognition of antigens expressed in tumour cells via the T-cell receptor, activated CD8+ T cells attack tumour cells and are therefore referred to as cytotoxic T cells (CTL)^20^. The PD-L1/PD-1 pathway has been demonstrated to inhibit the T-cell anti-tumour immune response, and PD-L1 preferentially co-stimulates IL-10 production in T cells. Upregulated PD-L1 expression in chemoresistant ovarian cancer cells might affect tumour-reactive T-cell function and PD-L1-induced IL-10 expression. IFN-γ and TNF-α, which are cytokines produced and secreted by inflammatory cells in the tumour microenvironment, are major stimulators of PD-L1 expression in tumour cells^21^. To determine if miR-424(322) affects T-cell function via PD-L1 expression inhibiting, we established the Skov3 (CP)/T-cell co-culture model. In IFN-γ- or TNF-α-treated Skov3 (CP) cells, IL-10 production was increased compared with the untreated cells (Fig. 4a, Supplementary Fig. 5A). Furthermore, the co-culture of T cells with IFN-γ- or TNF-α-pre-treated Skov3 (CP) cells increased the apoptosis of PD-L1+/CD8+ T cells compared with the T cells cultured with untreated Skov3 (CP) cells (Fig. 4b,c, Supplementary Fig. 5B,C). However, in Skov3 (CP) cells that overexpressed miR-424(322), the apoptosis of PD-L1+/CD8+ T cells induced by IFN-γ or TNF-α was completely abolished, and the IL-10 production was also inhibited (Fig. 4a–c, Supplementary Fig. 5A–C). The effect of anti-PD-L1 was similar to miR-424(322)-mediated apoptosis of PD-L1+/CD8+ T cells, which suggests that miR-424(322) repressed IFN-γ- or TNF-α-induced PD-L1-associated CD8+ T-cell apoptosis by blocking PD-L1. These findings also suggest that chemotherapy-induced immune-resistance could be reversed, in part by miR-424(322) overexpression. These findings indicate that the suppression of PD-L1 expression by increasing miR-424(322) expression might improve the therapeutic efficacy of chemoresistant tumours associated with PD-L1 overexpression.

### CD8+ T cells are required for miR-424(322) treatment

**Figure 5:**
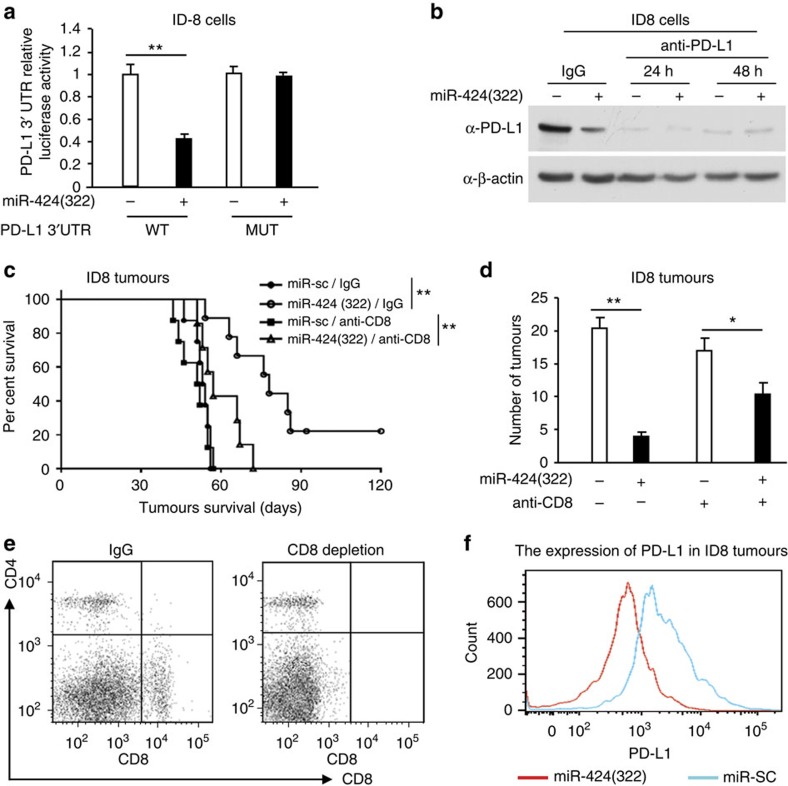
CD8+ T cells are required for the efficacy of miR-424(322) treatment in ID8 tumours. (**a**) The luciferase vectors that contained the mouse wild-type (WT) and mutant (MUT) PD-L1 3′-UTR regions were co-transfected into ID8 cells with miR-424(322) or scramble miRNA precursors (miR-Scr). The relative luciferase/*Renilla* activities were analysed in the cells 48 h after the transfection. *t-test*, **P≤0.01. The results represent the mean±s.e.m. from three independent experiments. (**b**) ID8 cells with stable overexpression of miR-424(322) were treated with anti-PD-L1 or IgG control for 24–48 h. PD-L1 protein levels were determined by western blotting. (**c**–**f**) ID8 cells (5 × 10^6^) with stable overexpression of miR-424(322) were injected into the syngeneic C57BL/7 mice followed by CD8+ blocking antibody (anti-CD8) treatment. (**c**) Kaplan–Meier survival analysis of tumour-bearing mice in different treatment groups. *t-test*, **P≤0.01. (**d**) The number of tumours was determined in different treatment groups. *t-test*, *P≤0.05, **P≤0.01. Bar graphs are shown as the mean±s.e.m. (*n*=12 mice per group). (**e**,**f**) At the time of necropsy, CD8 and PD-L1 expression was detected by flow cytometry. (**e**) FACS analysis of CD8 expression in ID8 tumours. CD8 expression was decreased in the anti-CD8-treated versus untreated mice. (**f**) FACS analysis of cell-surface PD-L1 expression in ID8 tumours. PD-L1 expression was decreased in the ID8 tumours with stable overexpression of miR-424(322).

We analysed the 3′-UTR of the mouse PD-L1 gene using the public database *microRNA.org* and determined that mouse PD-L1 was also a potential target of mouse miR-424(322) (Supplementary Fig. 6A). To confirm these targets, reporter constructs were generated that contained the putative binding site of the 3′-UTR regions of the PD-L1 gene or mutants of the putative sites of mouse miR-424(322) binding. The luciferase activity of PD-L1 wild-type constructs was inhibited by miR-424(322) overexpression (Fig. 5a) and induced by miR-424(322) inhibition (Supplementary Fig. 6B) in ID8 cells. Point mutations in the putative binding site abrogated the inhibition by miR-424(322) (Fig. 5a, Supplementary Fig. 6B), demonstrating that miR-424(322) specifically targeted the PD-L1 3′-UTR, likely through the putative binding sites. Western blotting analyses indicated that miR-424(322) overexpression in ID8 cells decreased the protein levels of PD-L1 (Fig. 5b).

To investigate the role of miR-424(322) in immune response regulation *in vivo*, we transplanted ID8 cells that stably overexpressed miR-424(322) into syngeneic C57BL/7 mice, followed by CD8+ blocking antibody (anti-CD8) treatment. The survival of the mice is shown in Fig. 5c. The mice with miR-424(322)-overexpressing tumours survived significantly longer than the mice in the miR-SC control group, whereas anti-CD8 treatment eliminated the miR-424(322)-mediated survival benefit. These findings suggest that CD8+ T cells are required for the efficacy of miR-424(322) function in ID8 tumours. Moreover, there was no effect of anti-CD8 treatment on the survival of mice that exhibited tumours without miR-424(322) overexpression (Fig. 5c). We also determined the number of tumours in C57BL/7 mice. As shown in Fig. 5d, miR-424(322) overexpression alone significantly decreased the number of tumours compared with miR-Sc in C57BL/7 mice, whereas anti-CD8 treatment inhibited the efficacy of miR-424(322). Fluorescence activating cell sorter (FACS) analysis indicated that the CD8 expression was lower in the anti-CD8-treated mice versus untreated mice (Fig. 5e), and the cell-surface PD-L1 expression was lower in the ID8 tumours that overexpressed miR-424(322) (Fig. 5f).

We subsequently investigated the role of endogenous miR-424(322) in tumour growth regulation *in vivo*. We injected ID8 cells into the syngeneic C57BL/7 mice, followed by anti-miR-424(322) treatment. Inhibition of miR-424(322) decreased the overall survival and increased the number of tumours in the C57BL/7 mice (Supplementary Fig. 6C,D). FACS analysis showed that cell-surface PD-L1 expression was lower in anti-miR-424(322)-treated ID8 tumours (Supplementary Fig. 6E).

### miR-424(322) regulates immunocyte production

**Figure 6:**
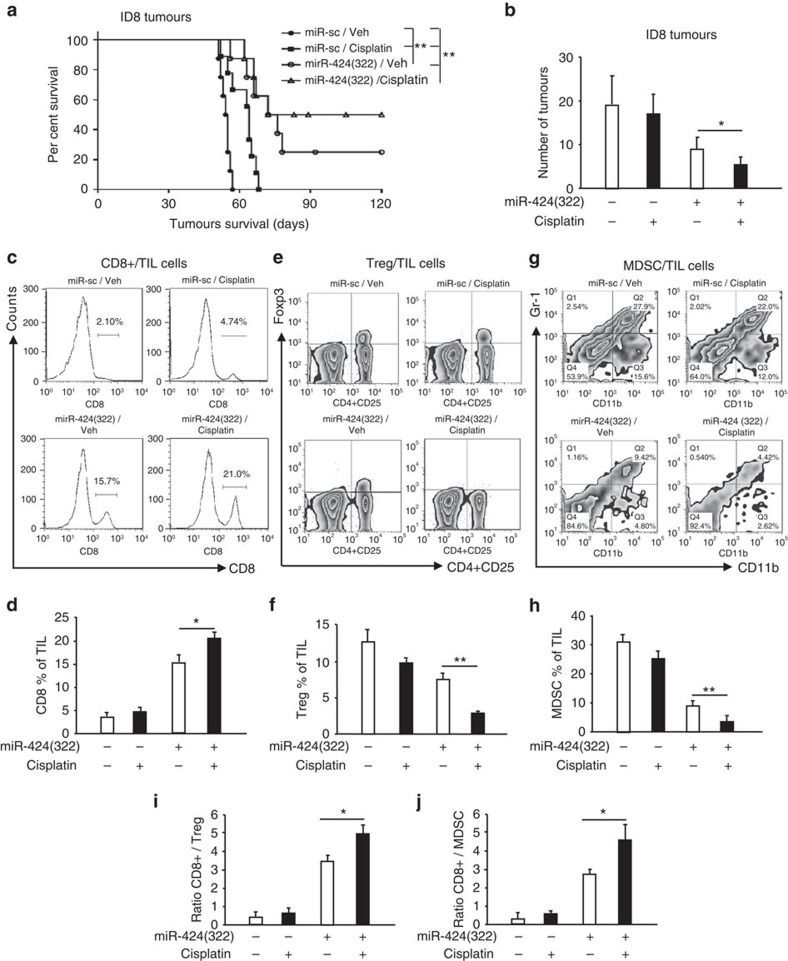
miR-424(322) enhances the efficacy of chemotherapy by regulating immunocyte production in ID8 tumours. ID8 cells (5 × 10^6^) with stable overexpression of miR-424(322) were injected into the syngeneic C57BL/7 mice followed by cisplatin or vehicle (Veh) treatment. (**a**) Kaplan–Meier survival analysis of tumour-bearing mice in different treatment groups. *t-test*, **P≤0.01. (**b**) The number of tumours was determined in different treatment groups. *t-test*, *P≤0.05. Bar graphs are shown as the mean±s.e.m. (*n*=12 mice per group). (**c**–**h**) CD8+ T cells, Treg cells and MDSC from tumours of ID8 tumour-bearing mice were isolated and counted by FACS analysis. (**c**,**d**) Percentages of CD8+ TILs (CD45+) infiltration of total leucocytes. (**e**,**f**) Changes in percentages of CD4+ CD25+ Foxp3+ Treg in TILs. (**g**,**h**) Representative of MDSCs gated on a CD45+ cell population are shown. Cisplatin treatment significantly increased the CD8+ T-cell population and decreased the MDSC and Treg-cell population in the C57BL/7 mice with miR-424(322)-overexpressing tumours. *t-test*, *P≤0.05, **P≤0.01. Bar graphs are shown as the mean±s.e.m. (*n*=12 mice per group). (**i**,**j**) The ratios of CD8+ T cells to Treg cells or MDSC from tumour of ID8 tumour-bearing mice were determined by FACS analysis. Cisplatin treatment significantly increased the ratio of CD8+ T cells to Treg cells or MDSC in the C57BL/7 mice with miR-424(322)-overexpressing tumours. *t-test*, *P≤0.05. Bar graphs are shown as the mean±s.e.m. (*n*=12 mice per group).

PD-L1 expression in the tumour microenvironment is associated with poor outcomes following chemotherapy in cancer patients^22^. Increased PD-L1 expression following chemotherapy and a subsequent decrease in cancer cell-specific T-cell activity might promote the development of immune response evasion in tumours^12^. Our previous studies demonstrated that the expression of PD-L1 was directly repressed by miR-424(322). To investigate the effect of miR-424(322) and cisplatin combination treatment in ovarian cancer, we injected ID8 cells that stably expressed miR-424(322) into syngeneic C57BL/7 mice, followed by cisplatin or vehicle (Veh) treatment. The survival rate of the mice is shown in Fig. 6a. The mice with miR-Sc-overexpressing tumours in the cisplatin treatment group lived slightly longer than the control group mice. However, the mice with miR-424(322)-overexpressing tumours in the cisplatin treatment group survived significantly longer than the mice in the miR-424(322)/Veh control group, which indicates that miR-424(322) might cooperate with cisplatin to suppress the tumour progression of ID8 tumours. miR-424(322) alone exhibited inhibitory effects on tumour growth, which indicates that miR-424(322) might function as a tumour suppressor. We subsequently determined the number of tumours in C57BL/7 mice and found a similar result. As shown in Fig. 6b, cisplatin treatment did not decrease the number of tumours in the C57BL/7 mice with miR-Sc-overexpressing tumours. However, cisplatin treatment significantly decreased the number of tumours in the C57BL/7 mice with miR-424(322)-overexpressing tumours.

Our previous studies demonstrated that CD8+ T cells are required for miR-424(322) to inhibit ID8 tumour growth. Using the same model, we examined the abundance of CD8+ T cells in ID8 tumours using a FACScan flow cytometer. As shown in Fig. 6c, cisplatin treatment had no effect on the CD8+ T-cell population in the C57BL/7 mice with miR-Sc-overexpressing tumours. However, cisplatin treatment significantly increased the CD8+ T cells (20%) compared with miR-424(322) (15%) or cisplatin (4%) in the C57BL/7 mice with miR-424(322)-overexpressing tumours, which indicates that miR-424(322) might activate the T-cell immune response during chemotherapy. Myeloid-derived suppressor cells (MDSC) and regulatory T (Treg) cells are major components of the immune-suppressive tumour microenvironment and have been correlated with chemoresistance^23^. Both cell types systematically expand in preclinical tumour models and promote T-cell dysfunction that favours tumour progression^23^. We also examined MDSC and Treg cells in ID8 tumours. As shown in Fig. 6d–h, cisplatin treatment did not increase or decrease the MDSC and Treg-cell population in the C57BL/7 mice with miR-Sc-overexpressing tumours. However, cisplatin treatment significantly decreased the MDSC and Treg-cell numbers in the C57BL/7 mice with miR-424(322)-overexpressing tumours, which indicates that miR-424(322) represses T-cell dysfunction during chemotherapy. The ratios of CD8+ T cells to Treg cells or MDSC in ID8 tumours were also determined. As shown in Fig. 6i,j, cisplatin treatment did not increase or decrease the ratios of CD8+ T cells to Treg cells or MDSC in the C57BL/7 mice with miR-Sc-overexpressing tumours. However, cisplatin treatment significantly increased the ratios of CD8+ T cells to Treg cells or MDSC in the C57BL/7 mice with miR-424(322)-overexpressing tumours. Taken together, these findings further demonstrate that miR-424(322) enhanced the efficiency of chemotherapy by regulating CD8+ T, MDSC and Treg-cell abundance in ID8 tumours.

### miR-424(322) activates CTLs and reduces regulatory cytokine secretions

**Figure 7:**
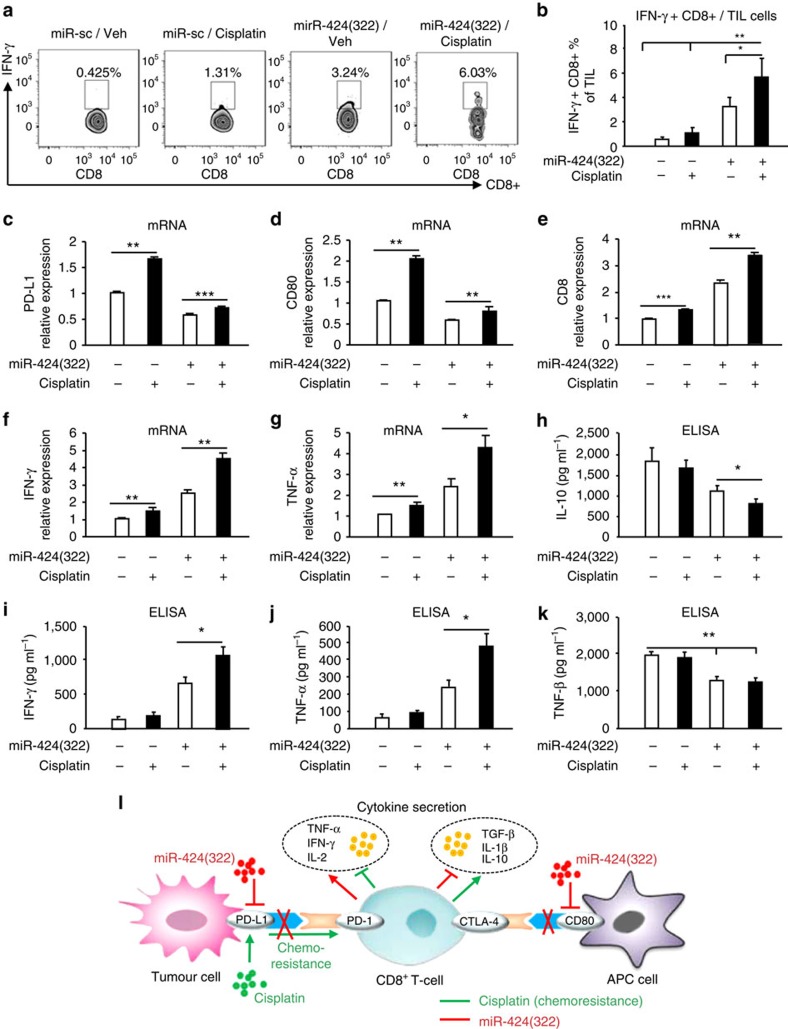
miR-424(322) enhances the efficacy of chemotherapy by activating cytotoxic T cells and reducing regulatory cytokine secretions in ID8 tumours. ID8 cells with stable overexpression of miR-424(322) were injected into syngeneic C57BL/7 mice, followed by cisplatin or vehicle (Veh) treatment. The T cells were harvested from regressing tumours and stained with various markers. (**a**,**b**) IFN-γ+/CD8+ T cells from tumour of ID8 tumour-bearing mice were isolated and counted. Cisplatin treatment significantly increased the number of IFN-γ+/CD8+ T cells in the C57BL/7 mice with miR-424(322) overexpressing tumours. *t-test*, *P≤0.05, **P≤0.01. Bar graphs are shown as the mean±s.e.m. (*n*=12 mice per group). (**c**–**g**) Relative expression levels of PD-1, PD-L1, CD80 and IFN-γ from tumour of ID8 tumour-bearing mice were determined via qRT-PCR assay. Cisplatin treatment significantly repressed the mRNA levels of PD-L1 and CD80 and increased the mRNA levels of CD8, TNF-α and IFN−γ in the C57BL/7 mice with miR-424(322)-overexpressing tumours. *t-test*, *P≤0.05, **P≤0.01, ***P≤0.001. Bar graphs are shown as the mean±s.e.m. (*n*=12 mice per group). (**h**–**k**) Circulating serum from C57BL/7 mice was assayed for IL-10, TNF-α, IFN-γ and TGF-β using a cytokine ELISA assay. Cisplatin treatment significantly inhibited the secretion of IL-10 and promoted the secretion of TNF-α and IFN-γ in the C57BL/7 mice with miR-424(322)-overexpressing tumours. *t-test*, *P≤0.05, **P≤0.01. Bar graphs are shown as the mean±s.e.m. (*n*=12 mice per group). (**l**) Schematic representation of the biological and functional interactions between PD-L1 and chemoresistance through the miR-424(322) regulatory cascade.

Antigen-specific cytotoxic CD8+ T cells play critical roles in immune responses to infectious pathogens^24^. IFN-γ production is a surrogate marker for CTL^24^. As shown in Fig. 7a,b, cisplatin treatment did not increase or decrease the number of IFN-γ+/CD8+ T cells in the C57BL/7 mice with miR-Sc-overexpressing tumours. However, cisplatin treatment significantly increased the number of IFN-γ+/CD8+ T cells by twofold in the C57BL/7 mice with miR-424(322)-overexpressing tumours, which suggests that miR-424(322) enhanced the efficacy of chemotherapy by CTL activation.

We demonstrated that PD-L1 and CD80 were directly regulated by miR-424(322), and miR-424(322) regulates T-cell cytokine secretions by blocking PD-L1 in a Skov3 (CP)/T-cell co-culture. We subsequently measured the mRNA levels of PD-L1, CD80, CD8, TNF-α and IFN-γ via RT-PCR in ID8 tumours. As shown in Fig. 7c,d, the mRNA levels of PD-L1 and CD80 were significantly increased following treatment with cisplatin alone in ID8 tumours. In contrast, the effects of cisplatin on PD-L1 and CD80 expression were slightly increased when miR-424(322) was overexpressed (lanes 3 and 4) compared with vector control (lanes 1 and 2), which suggests that miR-424(322) enhanced the efficacy of chemotherapy by reducing PD-L1 and CD80 expression *in vivo*. Furthermore, the mRNA levels of CD8, TNF-α and IFN−γ were increased following treatment with cisplatin alone in ID8 tumours. In contrast, the effects of cisplatin on CD8, TNF-α and IFN−γ were enhanced when miR-424(322) was overexpressed (lanes 3 and 4) compared with vector control (lanes 1 and 2) (Fig. 7e–g). T-cell cytokine secretions in the serum of C57BL/7 mice were subsequently measured. As shown in Fig. 7h–k, cisplatin treatment did not inhibit or induce the secretion of IL-10, TNF-α, IFN-γ and TGF-β in the C57BL/7 mice with miR-Sc-overexpressing tumours. However, cisplatin treatment significantly inhibited the secretion of IL-10 and induced the secretion of TNF-α and IFN-γ in the C57BL/7 mice with miR-424(322)-overexpressing tumours, which suggests that miR-424(322) enhanced the efficacy of chemotherapy by reducing the secretion of regulatory cytokines. Taken together, these findings indicate that miR-424(322) might improve chemotherapy-induced T-cell immune evasion through PD-L1 signalling (Fig. 7l).

## Discussion

Immune checkpoint blockade with antibodies that target CTLA-4 and the PD-1/PD-L1 pathway has been a promising strategy in the treatment of various malignancies^25^. PD-L1/PD-1 and CD80/CTLA-4 interactions inhibit CD8+ cytotoxic T-lymphocyte proliferation, survival and effector functions of tumour-infiltrating T cells^11^. CTLA-4 and PD-1/PD-L1 blockade would most likely be used as a novel immune checkpoint for blocking antibodies in initial clinical investigations^26^. Ipilimumab (anti-CTLA-4 antibody) and pembrolizumab (anti-PD-1 antibody) were recently approved by the US Food and Drug Administration^27^. To increase the number of patients who benefit from immune checkpoint blockade, CTLA-4 and PD-1/PD-L1 antibodies are being combined and would be administered in combination with other anticancer agents such as chemotherapy, targeted therapy, radiotherapy and other immunotherapies^26^. Many of these combinatorial approaches are founded on functional interactions; however, the related mechanisms that regulate the physiological and pathological conditions of tumours remain unclear.

miRNAs post-transcriptionally modulate gene expression by binding to the 3′-UTR^13^. Previous studies demonstrated that a class of miRNAs plays a pivotal role in the regulation of the host immune response. miR-200 leads to CD8+ T-cell immunosuppression via negative regulation of PD-L1 (ref. 15). miR-34a targets PD-L1 and functions as a potential immunotherapeutic target in acute myeloid leukaemia^12^. A MRX34 clinical trial is currently underway at MD Anderson Cancer Center^28^,^29^. A mouse study demonstrated that MRX34, an investigational drug that mimics the tumour-suppressing abilities of miR-34, increased CD8+ cells when combined with radiotherapy^28^,^29^. In our studies, analysis of the 3′-UTR of the PD-L1, PD-1, CD80 and CTLA-4 genes identified that PD-L1 and CD80 are potential targets of the miR-15a/15b/16/195/424/497/503 family. However, only miR-424(322) was inversely correlated with PD-L1, PD-1, CD80 and CTLA-4 expression in the clinical ovarian cancer data set. High expression levels of miR-424(322) were positively correlated with the PFS of ovarian cancer patients. Moreover, mechanistic investigations demonstrated that miR-424(322) decreased PD-L1 expression through direct binding to the 3′-UTR in the human and mouse genes. Therefore, our studies indicated a novel mechanism by which miR-424(322) influences the PD-L1/PD-1 signalling pathway.

Chemotherapy is a major treatment modality for ovarian cancer. However, chemoresistance is a clinical problem that compromises the efficiency of treatment and ultimately results in treatment failure^5^. Recent studies have demonstrated an upregulation of PD-L1 expression in cancer cells by chemo-preventive agents and a resulting decrease in tumour-specific T-cell activity that potentially promotes immune evasion^12^. There is a potential link between chemotherapy and immunoresistance. Herein, we demonstrated that low miR-424(322) levels and high PD-L1 expression were strongly associated with a chemoresistant phenotype in ovarian cancer cells and tumour tissues. Restoration of miR-424(322) expression in ovarian cancer chemoresistant cells resulted in a decrease in PD-L1 expression. miR-424(322) repressed IFN-γ-induced, PD-L1-associated CD8+ T-cell apoptosis and regulated T-cell cytokine secretions by blocking PD-L1 in ovarian cancer chemoresistant cells and a T-cell co-culture model. Furthermore, CD8+ T cells are required for the efficacy of miR-424(322) treatment in ovarian cancer. These findings indicate that endogenous miR-424(322) plays an important role in the development of immune evasion in chemoresistant ovarian cancer. The suppression of PD-L1 expression might be possible by increasing endogenous miR-424(322) expression, thereby improving the therapeutic index of the chemotherapy-based immune resistance.

PD-L1 blockade restored immune suppression and led to the expansion of TILs^30^. An increase in the number of CD4 and CD8+ cells was associated with increased CD8/Treg and CD4/Treg ratios^31^. This increase was associated with a moderate clinical response, and molecular evidence indicated functional activation of TILs. A parallel increase in MDSC contributed to the tumour immune-suppressive environment^32^. In this study, miR-424(322) in combination with cisplatin was very effective. This effect is likely a result of the downregulation of high PD-L1 expression by miR-424(322) in ID8 tumour cells and tumour-derived myeloid cells. The present findings suggest that tumours that lack TILs might have PD-L1-mediated suppression, which leads to the depletion of TILs. Our finding regarding the high frequency of Treg cells and MDSC in tumours that lack TILs is consistent with an association between Treg cells and a poor outcome in human ovarian cancer. Although miR-424(322) therapy resulted in moderately increased levels of TIL infiltration and proliferation, the Treg and MDSC levels were not dramatically reduced following miR-424(322) treatment alone. In this study, the combination of miR-424(322) and cisplatin indicated that miR-424(322) enhanced the efficacy of chemotherapy by regulating CD8+ T, MDSC and Treg-cell productions, activating CTL and reducing the secretion of regulatory cytokines in ID8 tumours.

CD80, which binds to the CTLA-4 receptor, is expressed on DCs^10^. CTLA-4 downregulates CD80 and CD86 on antigen presenting cells (APCs)^33^. Our studies demonstrated that CD80 is a target of miR-424(322) in humans, but not in mice. Overexpression of miR-424(322) in human DCs decreased the protein levels of CD80, which suggests that miR-424(322) might also regulate the immune response by blocking the CTLA-4 immune checkpoint signalling pathway.

We identified a novel mechanism by which miR-424(322) influences the PD-L1/PD-1 and CD80/CTLA-4 immune checkpoint signalling pathways. Restoration of miR-424(322) expression could reverse chemoresistance by T-cell immune response activation via blocking the PD-L1 immune checkpoint *in vitro* and *in vivo*. This synergistic effect of chemotherapy and immunotherapy was accompanied by a proliferation of functional cytotoxic CD8+ T cells and inhibition of MDSC and Treg cells. This study provides, compelling evidence that suggests a targeting strategy involving the functional interaction between miR-424(322) and the PD-L1/PD-1 immune checkpoint signalling to treat chemoresistant ovarian cancer.

## Methods

### Cell culture and reagents

The human ovarian cancer cell lines OVCAR-3, Skov3 and Skov3 (CP) were purchased from the Cell Bank of the Chinese Academy of Science. Cells were maintained in a medium of RPMI 1640 supplemented 10% FBS and 1% penicillin/streptomycin and were maintained in a 5% CO2 incubator at 37 °C. ID8, a cell line that was derived from spontaneous *in vitro* malignant transformation of C57BL/6 mouse ovarian surface epithelial cells was kindly provided by Dr Chen (Department of Gynaecology and Obstetrics, Tongji Hospital). Cells were maintained in DMEM, supplemented with 2 mmol l^−1^ L-glutamine (Sigma), 5% foetal bovine serum (Atlanta Biologicals), and 100 U ml^−1^ penicillin and 100 μg ml^−1^ streptomycin (Sigma). All cell lines were tested and free of mycoplasma contamination (MycoAlert Mycoplasma Detection Kit, Lonza). PLe-miR-SCR lentivirus vector and pLe-miR-424(322) were purchased from Open Biosystems, AL, USA. Cells were infected with pLe-miR-SCR or pLe-miR-424 followed by selection with FACS. has-miR-424(322) mimics and vector controls were purchased from Guangzh Ribobio, China.

### Tissue samples

To determine the correlation between miR-424(322) and PD-L1/CD80 in primary ovarian tumour samples, we examined previously described set of tissues that contained 42 ovarian cancer tumour specimens from the tissue bank of Nanjing Medical University^34^. All tumour ovarian tissues were collected immediately following surgical removal and snap-frozen in liquid nitrogen. The clinical characteristics of the ovarian cancer patients are listed in Supplementary Table 1. All patients provided written informed consent and the experimental procedures were approved by the Institutional Review Board of Nanjing Medical University. PFS was calculated from the time of surgery to the time of progression or recurrence. PFS>6 months was defined as sensitivity to the last platinum-based chemotherapy, and PFS<6 months was defined as resistant to the last platinum-based chemotherapy.

### Cell transfection with pre-miRNA precursors or anti-miRNA inhibitors

hsa-miR-424 mimics and hsa-miR-SC controls were transfected into cells using Lipofectamine 2000 (Invitrogen, CA, USA) according to the manufacturer's instructions. Protein lysates and total RNA were collected 48 h after the transfection. The expression levels of the miRNAs were verified by stem-loop qRT-PCR analysis.

### qRT-PCR

Total RNA was isolated using TRIzol reagent (Life Technologies, Carlsbad, CA, USA) according to the manufacturer's instructions. cDNAs were synthesized with TaqMan Reverse Transcription Reagents (Life Technologies). The expression levels of the miRNAs were analysed using TaqMan MicroRNA Assay Kits (Applied Biosystems, Foster City, CA) specific for hsa-miR-424 and PD-L1 mRNAs. The fold changes were determined using the comparative cycle threshold method (method (2^−ΔΔCT^)). All experiments were performed in triplicate^35^.

### Luciferase reporter assay

PD-L1 and CD80 3′-UTR regions that contained predicted miR-424-binding sites were amplified by PCR from genomic DNA. The PCR fragments were inserted into the UTR downstream of the luciferase gene in the pMIR-reporter luciferase vector (Ambion). Mutation of the putative miR-424(322) target sequence within the PD-L1 and CD80 3′-UTR were generated using QuickChange Site-Directed Mutagenesis Kit (Stratagene). Luciferase reporter plasmid, β-galactosidase (β-gal) plasmid, and pre-miR-424 and negative control precursors were co-transfected into cells using Lipofectamine 2000. Luciferase activities were measured 48 h after transfection using β-gal for normalization^36^.

Human: PD-L1-3′-UTR-F: 5′- GCGCTCGAGGGAGACGTAATCCAGCATT -3′; PD-L1-3′-UTR-R: 5′- AATGCGGCCGCCACCTTACAAATACTCCAT -3′

Mouse: PD-L1-3′-UTR-F: 5′- GCGCTCGAGCTATGATCACTCTCCAGAT -3′; PD-L1-3′-UTR-R: 5′- AATGCGGCCGCAAAAAAAGAAAAACAAATG -3′

Human: CD80-3′-UTR-F: 5′- GCGCTCGAGGCCAGAACCCAGATTTCCT -3′; PD-L1-3′-UTR-R: 5′- AATGCGGCCGCCCTCTCTGCCCTACACTGAGA -3′

### Western blot analysis

Western blot assays were conducted in Skov3, ID8 and DCs cells as indicated. The cells were pelleted and lysed in buffer (50 mmol l^−1^ HEPES, pH 7.2, 150 mmol l^−1^ NaCl, 1 mmol l^−1^ EDTA, 1 mmol l^−1^ EGTA, 1 mmol l^−1^ DTT and 0.1% Tween 20) supplemented with a proteaseinhibitor cocktail (Roche Diagnostics). The antibodies used for western blots were: PD-L1 (Abcam, ab58810) and CD80 (Abcam, ab64116). All the primary antibodies were used at 1:1,000 dilutions and secondary antibodies at 1:5,000 dilutions. Uncropped scans of the immunoblots are shown in Supplementary Figs 7–10.

### Blood DCs isolation

Blood DCs were isolated from healthy human donors using a Human Blood Dendritic Cell Isolation Kit II (Miltenyi Biotec, No. 130-091-379) following the manufacturer's instruction.

### Skov3 (CP)/T-cell co-culture model

Peripheral blood mononuclear cells (PBMCs) from healthy human donors were isolated using Lymphoprep density gradient centrifugation (Accurate Chemical). PBMCs were plated at a density of 2 × 10^6^ per well in six-well plates and stimulated with Skov3 (CP) cell lysate, anti-CD3e (10 μg ml^−1^) and anti-CD28 (2 μg ml^−1^) for 48 h to promote T-cell activation. The T-cell activation protocol was provided by eBioscience (http://www.ebioscience.com/cell-type/t-cells.htm). Skov3 (CP) cells were transfected with miR-424(322) mimics or miR-SC controls for 48 h. Stimulated PBMCs were subsequently harvested and purified by Lymphoprep density gradient centrifugation, and co-cultured with the Skov3 (CP) cells at a 10:1 ratio for 16 h. The Skov3 (CP) cells were then sorted by FACS. The relative expression levels of PD-L1 in the Skov3 (CP) cells were determined by qRT-PCR assay. Co-culture media were assayed for TNF-α, IFN-γ, IL-2, IL-10, IL-1β and TGF-β using a cytokine enzyme-linked immunosorbent assay (ELISA) assay.

### T-cell apoptosis assay

PBMCs were isolated from healthy human donors using Lymphoprep density gradient centrifugation. PBMCs were plated at a density of 2 × 10^6^ per well in six-well plates and stimulated with Skov3 (CP) cell lysate, anti-CD3e (10 μg ml^−1^) and anti-CD28 (2 μg ml^−1^) for 48 h to promote T-cell activation. The Skov3 (CP) cells were first exposed to IFN-γ or TNF-α for 24 h, and the cell lysates were assayed for IL-10 using a cytokine ELISA assay. Stimulated PBMCs were subsequently harvested and purified by Lymphoprep density gradient centrifugation, and then co-cultured with mitomycin C-treated Skov3 (CP) cells at a 10:1 ratio for 16 h. Ten micrograms per millilitre anti-PD-L1 were added to the indicated wells to examine PD-L1-specific CD8+ T-cell apoptosis. The PBMCs were subsquently harvested and stained with PE-conjugated PD-L1, Alexa Fluor 488-conjugated annexin V and APC-conjugated CD8 antibodies. PD-L1-dependent, CD8+ T-cell apoptosis was calculated as the percentage of annexin V+ cells in the gated PD-L1+/CD8+ population^12^.

### Syngeneic orthotopic ovarian cancer model

The model was slightly modified as previously described^37^,^38^. ID8 cells were cultured, harvested and suspended in PBS. A total of 0.5 ml that contained 5 × 10^6^ cells was s.c. injected into the peritoneal cavity of 6- to 8-week-old female C57BL/7 mice (Chinese Academy of Sciences at Beijing, China). Mice were housed in groups of five under specific pathogen-free conditions with unlimited access to food and water. Ten days after injection, the mice were treated with cisplatin (7.5 mg kg^−1^, once). Tumour growth and survival were assessed. Tumours tissues were used for characterization of MDSC, Treg and CD8+ T-cell memory subsets. All the experimental protocols and animal care were approved by the Institutional Review Board of Tongji Medical college, Huazhong University of Science and Technology.

### Enzyme-linked immunosorbent assay

The production of T-cell cytokines was detected with a cytokine ELISA using the human or mouse ELISA kit (ExCell Bio, Shanghai, China) as previously described^12^. Briefly, 96-well plates were coated with cytokine capture antibodies overnight at 4 °C. After washing three times with the wash buffer, the wells were blocked with blocking buffer for 1 h at room temperature, and subsequently washed three times with wash buffer. One hundred microliters of cell culture supernatant were added to the appropriate wells and incubated for 2 h at room temperature. After washing, 100 μl of detection antibody were added and then incubated for 1 h at room temperature, followed by three washes. One hundred microliters of diluted streptavidin peroxidase were added, followed by three washes. TMB substrate (3,3′5,5′-tetramethylbenzidine; Sigma, USA) was added, and the reaction was stopped by the addition of 25 μl of 1.25 M sulphuric acid. Enzyme activity was measured at 450 nm using an ELISA reader.

### Analysis of 2011 TCGA data set

A normalized mRNA expression data set for ovarian cancer^39^ was downloaded from the cBioPortal for cancer genomics and used to evaluate PFS and PD-L1, CD80, PD-1, CTLA-4 and miR-424(322) transcript levels. This data set includes mRNA profiles for 489 ovarian tumour samples. Spearman's correlation coefficient was calculated for these transcripts for all primary tumour samples. Differences were considered significant at *P*<0.05.

### Statistical analysis

All statistical analyses were performed using EXCEL 2010 (Microsoft, USA). The results were reported as mean±s.d. Statistical analyses were conducted using Student's *t*-test and Pearson Correlation Coefficient. *P*<0.05 were considered statistically significant.

## Additional information

**How to cite this article:** Xu, S. *et al*. miR-424(322) reverses chemoresistance via T-cell immune response activation by blocking the PD-L1 immune checkpoint. *Nat. Commun.* 7:11406 doi: 10.1038/ncomms11406 (2016).

## Supplementary Material

Supplementary Information Supplementary Figures 1-10 and Supplementary Table 1
