# Supplementary material for: miR-424(322) reverses chemoresistance via T-cell immune response activation by blocking the PD-L1 immune checkpoint
Source: Nat Commun. 2016 May 5;7:11406. doi: 10.1038/ncomms11406 (PMC4858750; doi:10.1038/ncomms11406)
Supplement: Supplementary Information — Supplementary Figures 1-10 and Supplementary Table 1 [file ncomms11406-s1.pdf]

A

Human CD274 3'-UTR

526 AUAUUGUAGUAGAUGUUACAAUUUUGUCGCCAAACUAAACUUGCUGCUUAAUGAUUUGCUCACAUCUAGUAAAAC 600
601 AUGGAGUAUUUGU 613

miR-503
miR-497
miR-424
miR-195 ← miRNA family
miR-16
miR-15b
miR-15a

B

Human CD80 3'-UTR

401 CAUAUAUAUGUCAGGCAAAGUGCUGCUGGAAGUAGAAUUUGUCCAAUAACAGGUCAACUUCAGAGACUAUCUGA 474

miR-497
miR-424
miR-195 ← miRNA family
miR-16
miR-15b
miR-15a

486 AGAGUAGAAGAUUUUAUGCUGCUUUUACAAAAGCCCAAUGUAAUGCAUAGGAAGUAUGGCAUGAACAUCUUUAGG 562

miR-497
miR-424
miR-195 ← miRNA family
miR-16
miR-15b
miR-15a

601 CAUUUUUUUCAUUGUGUUCUCUAUUGCUGCUCUCUCACUCCCCCAUGAGGUACAGCAGAAAGGAGAACUAUCC 672

miR-497
miR-424
miR-195 ← miRNA family
miR-16
miR-15b
miR-15a

1201 UUAAAGCCUUUAGAGCCAGCCCAUGGCUUUAGCUACCUCACUAUGCUGCUUCACAACCUUGCUCCUGUG 1270

miR-497
miR-424
miR-195 ← miRNA family
miR-16
miR-15b
miR-15a

Supplementary Figure 1. PD-L1 and CD80 are identified as potential targets of the miR-15a/15b/16/195/424/497/503 family using the public database microRNA.org. (A) PD-L1 was potential target of miR-15a/15b/16/195/424/497/503 family (B) CD80 was potential target of miR-15a/15b/16/195/424/497/503 family.

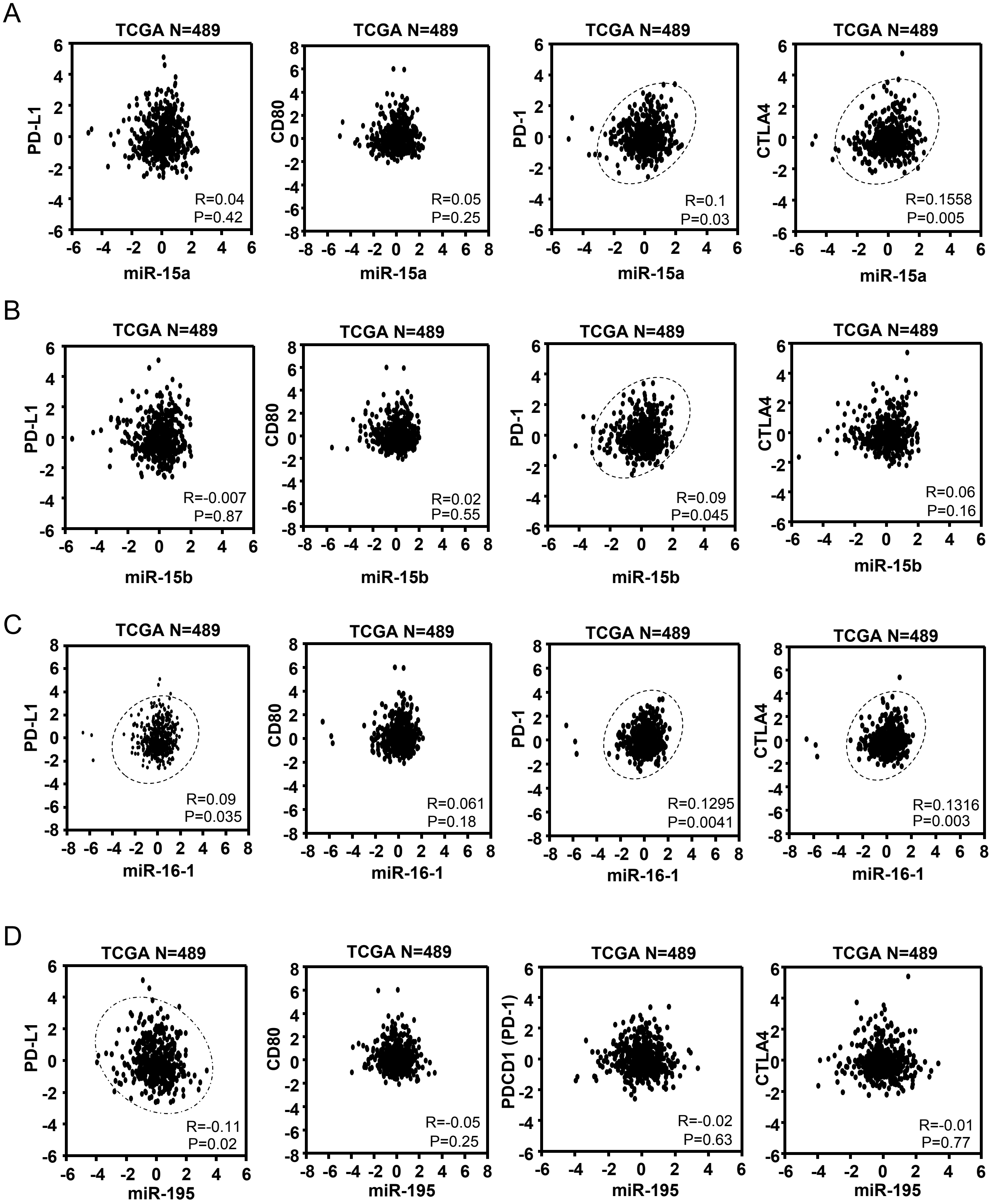
A
TCGA N=489
PD-L1
miR-15a
R=0.04
P=0.42
TCGA N=489
CD80
miR-15a
R=0.05
P=0.25
TCGA N=489
PD-1
miR-15a
R=0.1
P=0.03
TCGA N=489
CTLA4
miR-15a
R=0.1558
P=0.005
B
TCGA N=489
PD-L1
miR-15b
R=-0.007
P=0.87
TCGA N=489
CD80
miR-15b
R=0.02
P=0.55
TCGA N=489
PD-1
miR-15b
R=0.09
P=0.045
TCGA N=489
CTLA4
miR-15b
R=0.06
P=0.16
C
TCGA N=489
PD-L1
miR-16-1
R=0.09
P=0.035
TCGA N=489
CD80
miR-16-1
R=0.061
P=0.18
TCGA N=489
PD-1
miR-16-1
R=0.1295
P=0.0041
TCGA N=489
CTLA4
miR-16-1
R=0.1316
P=0.003
D
TCGA N=489
PD-L1
miR-195
R=-0.11
P=0.02
TCGA N=489
CD80
miR-195
R=-0.05
P=0.25
TCGA N=489
PDCD1 (PD-1)
miR-195
R=-0.02
P=0.63
TCGA N=489
CTLA4
miR-195
R=-0.01
P=0.77

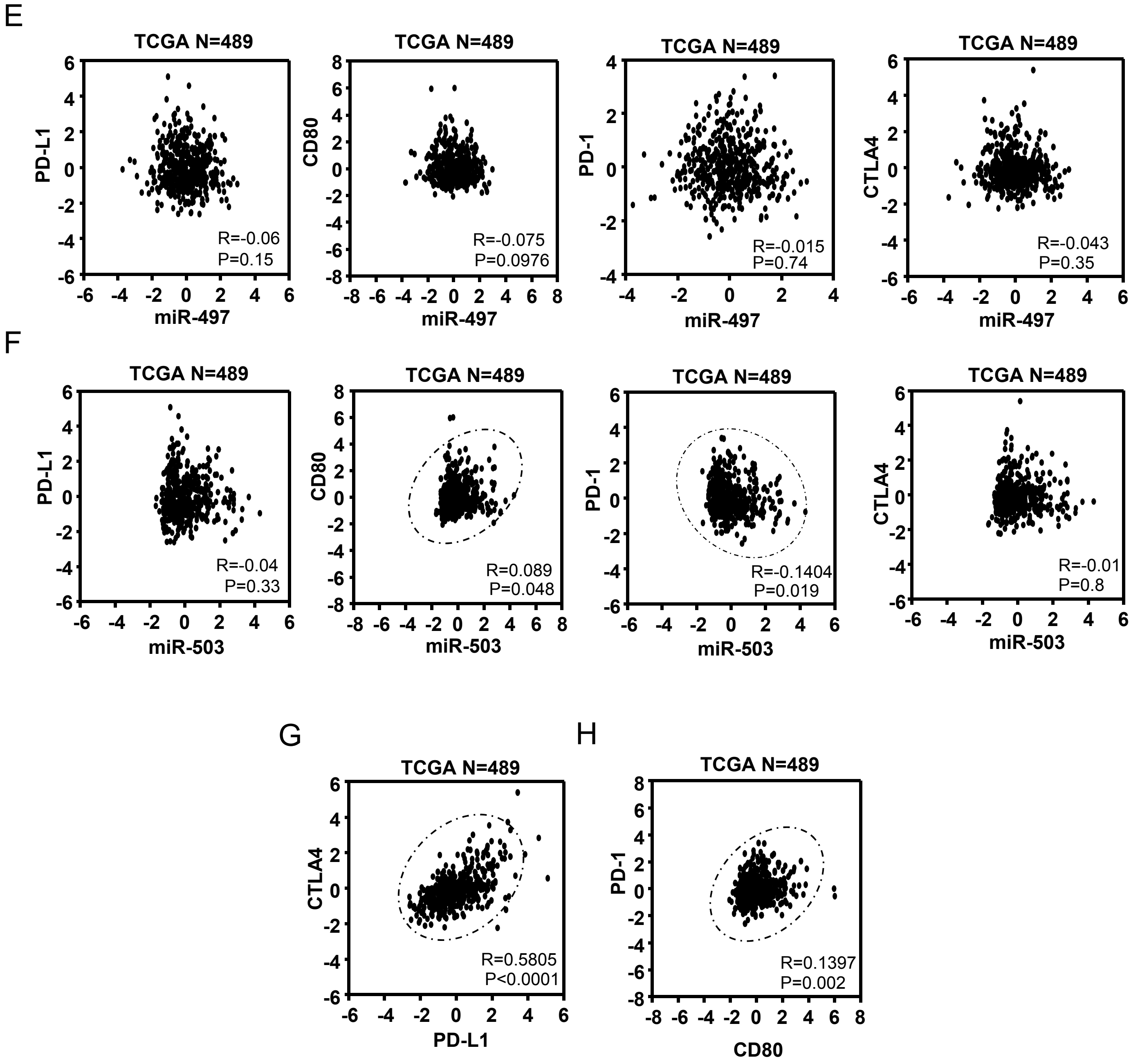

Supplementary Figure 2. Relevance of PD-L1/PD-1/CD80/CTLA-4 and the miR-15a/15b/16/195//497/503 family in a TCGA dataset composed of 489 ovarian cancer patient samples. (A-F) The relevance of PD-L1/PD-1/CD80/CTLA-4 and miR-15a/15b/16/195/497/503 family in a 2011 TCGA dataset. (G) PD-L1 expression levels were positively correlated with CTLA-4 expression levels in human ovarian cancer (r = 0.5805, p < 0.0001). (H) CD80 expression levels were positively correlated with PD-1 expression levels in human ovarian cancer (r = 0.1397, p = 0.002).

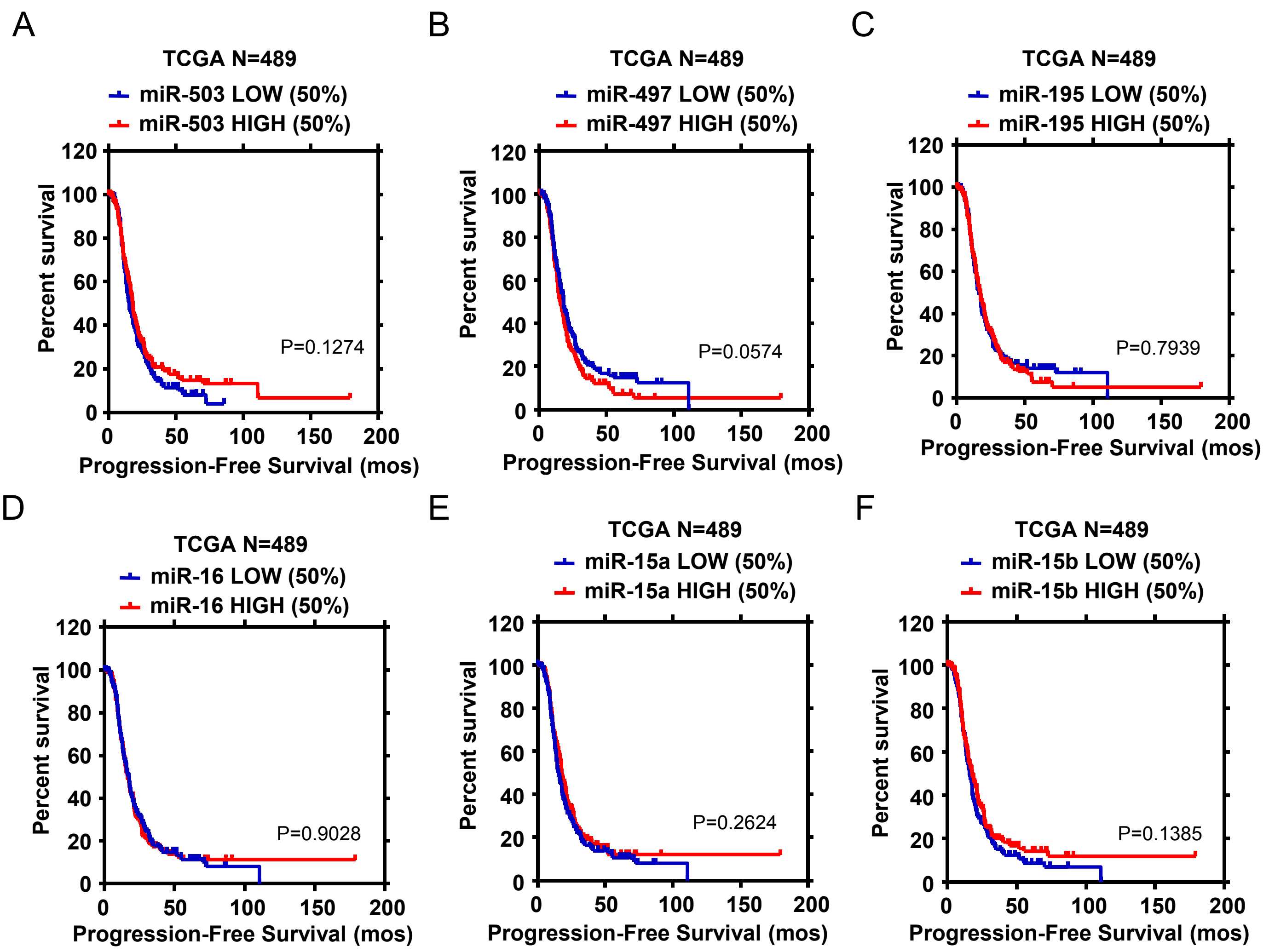

Supplementary Figure 3. Disease progression-free survival (PFS) was evaluated for miR-15a/15b/16/195/424/497/503 family using Kaplan-Meier analysis in the TCGA dataset. (A-F) Kaplan-Meier analysis was conducted to evaluate the correlation between the disease PFS of the patients and the expression of miR-15a/15b/16/195/497/503 family members in tumour samples. The miR-15a/15b/16/195/497/503 expression was used to assign the samples to high (upper 50th percentile) or low (lower 50th percentile) groups.

A

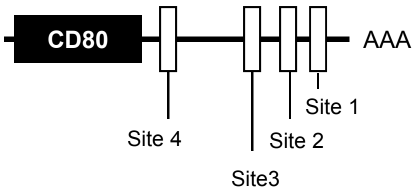

Site 1 (pos. 414-430)

```
ugucAGGCA--AAGUGCUGCUg
aaguUUUGUACUUAACGACGAc
```

hsa-miR-424(322)

Site 4 (pos. 1235-1250)

```
cuACCUCACUAUGCUGCUu
uuUGUACUUA-ACGACGAc
```

hsa-miR-424(322)

Supplementary Figure 4. miR-424(322) targeting sites in 3'-UTRs of CD80 are shown.
(A) The miR-424(322) targeting sites in 3'-UTRs of human CD80 are shown.

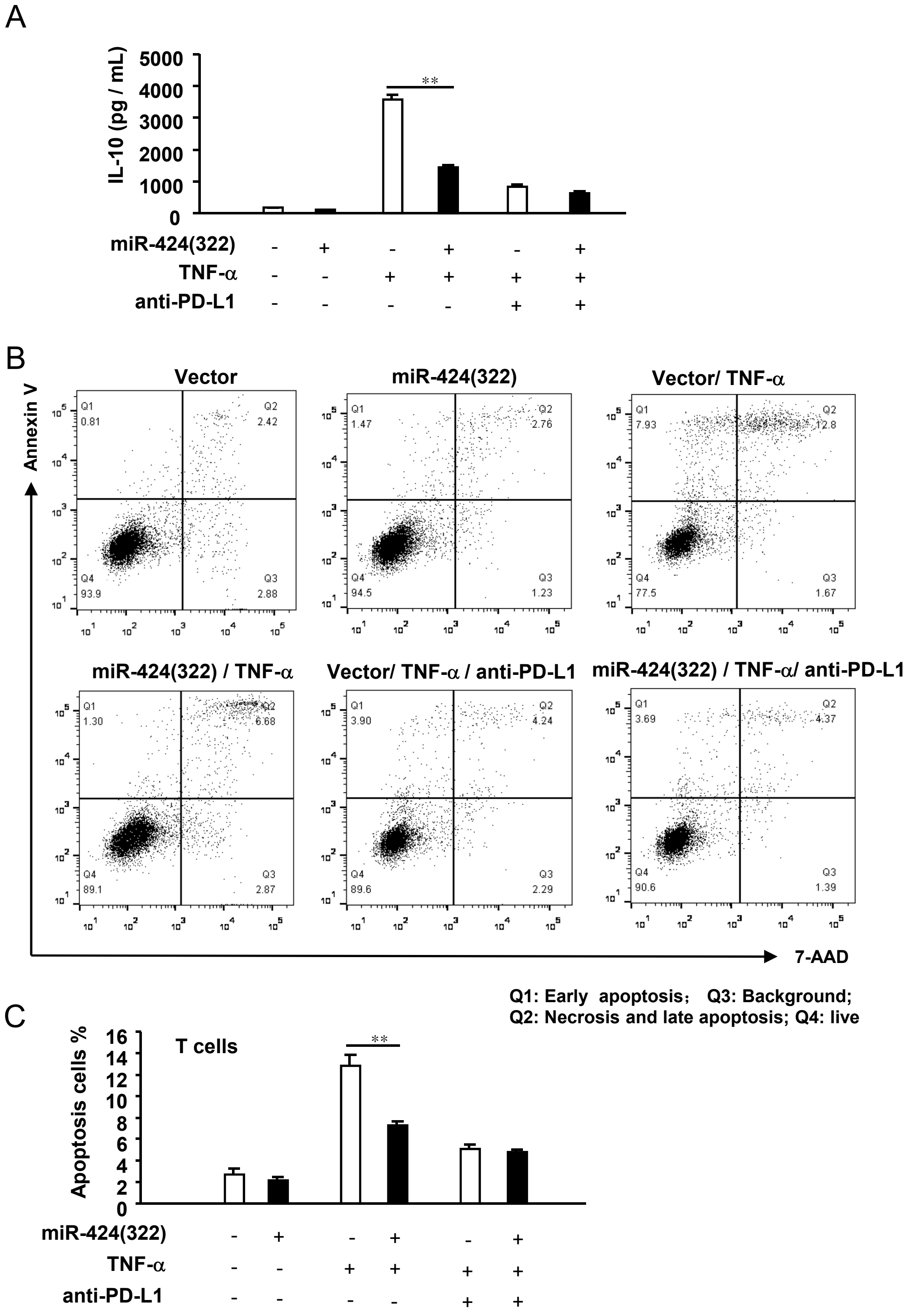

Supplementary Figure 5. miR-424(322) influences TNF-α induced PD-L1-associated CD8+ T cells apoptosis in a Skov3 (CP)/T cells co-culture model. Skov3 (CP) cells with stable overexpression of miR-424(322) or miR-Src were first exposed to TNF-a for 24 h in the presence or absence of anti-PD-L1. (A) Culture media were assayed for IL-10 using a cytokine ELISA assay. ** $p \leq 0.01$. The results represent the mean ± SEM from three independent experiments. (B-C) T cells were subsequently co-cultured with mitomycin C-treated Skov3 (CP) cells for 24 h. T cells were sorted by FACS, stained with PE anti-PD-L1, Alexa Fluor 488 anti-annexin V and APC anti-CD8 and analysed by flow cytometry for T-cell apoptosis in the PD-L1+/CD8+ population. ** $p \leq 0.01$. The results represent the mean ± SEM from three independent experiments, and the densitometric level of the apoptosis ratio is shown.

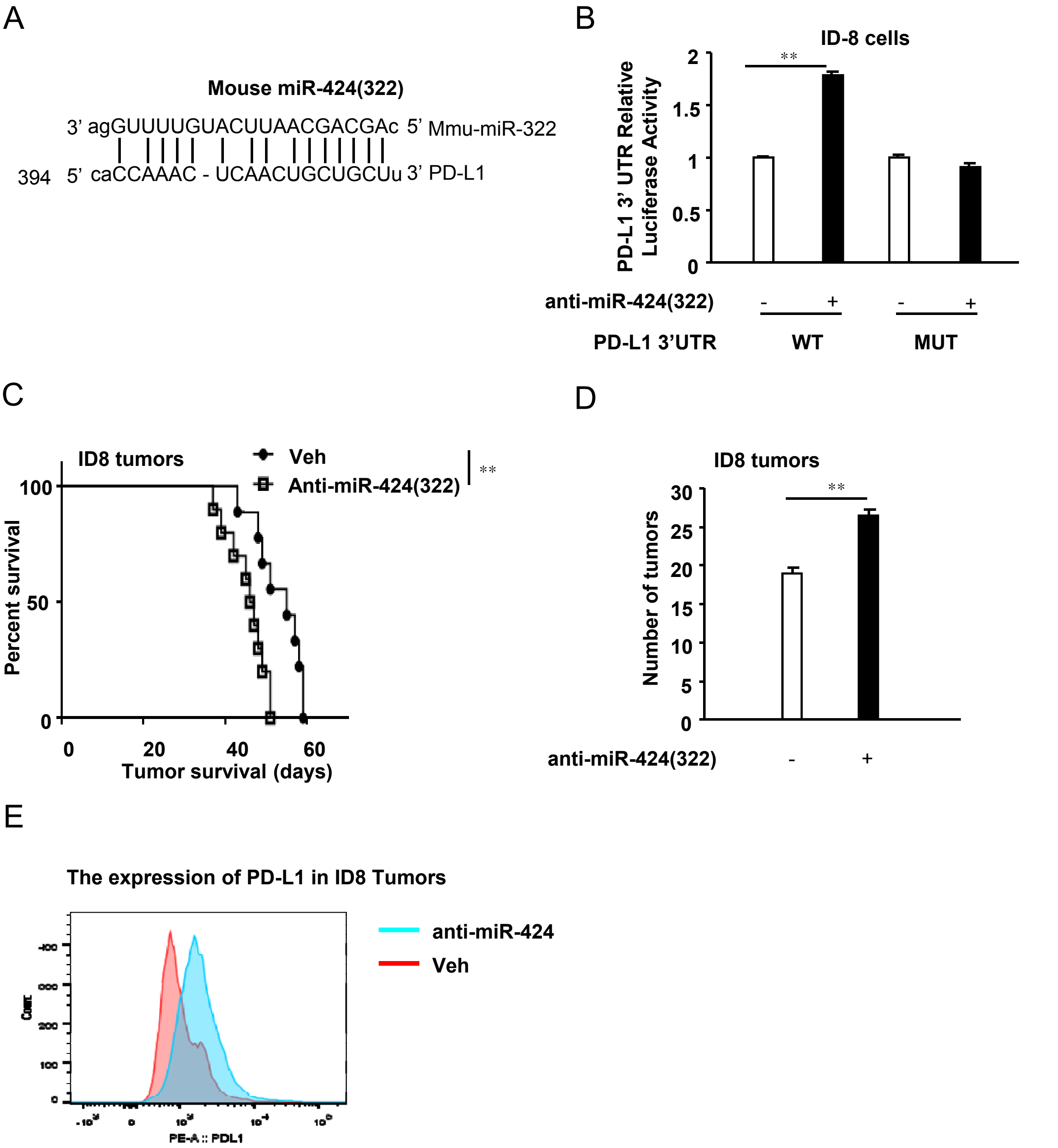

Supplementary Figure 6. miR-424(322) inhibits ID8 tumour growth. (A) The miR-424(322) targeting sites in the 3'-UTR of mouse PD-L1 are shown. (B) The luciferase vectors that contained the mouse wild-type (WT) and mutant (MUT) PD-L1 3'-UTR regions were co-transfected into ID8 cells with anti-miR-424(322) or vector control. The relative luciferase/*Renilla* activities were analysed in the cells 48 h after the transfection. The results represent the mean ± SEM from three independent experiments. ** $p \leq 0.01$. (C-E) ID8 cells (5×106) were injected into the syngeneic C57BL/7 mice, followed by miR-424(322) inhibitor (anti-miR-424(322)) or vehicle (Veh) treatment. (C) Kaplan-Meier survival analysis of tumour-bearing mice in different treatment groups. ** $p \leq 0.01$. (D) The number of tumours was determined in different treatment groups. ** $p \leq 0.01$. Bar graphs are shown as the mean ± SEM (n = 12 mice/group). (E) FACS analysis of cell-surface PD-L1 expression in ID8 tumours. PD-L1 expression was increased in anti-miR-424(322) treated ID8 tumours.

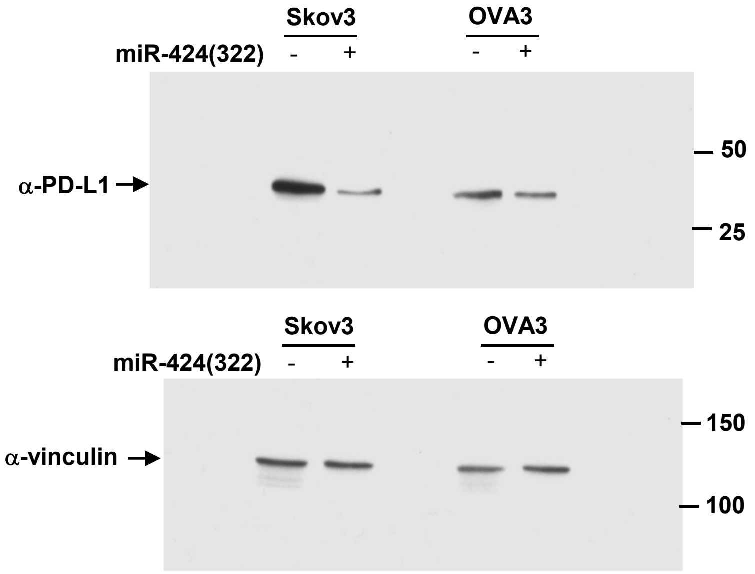

Supplementary Figure 7. Original blots for western blots in Figure 2E. miR-424(322) overexpression in Skov3 and OVA3 human cancer cells decreased the protein levels of PD-L1.

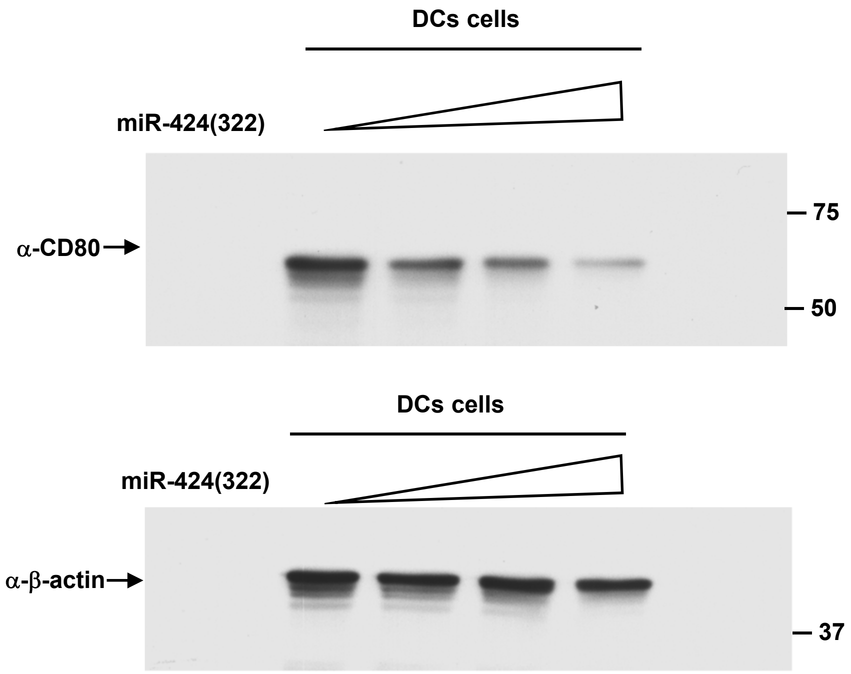

Supplementary Figure 8. Original blots for western blots in Figure 2F. miR-424(322) overexpression in DC cells decreased the protein levels of CD80.

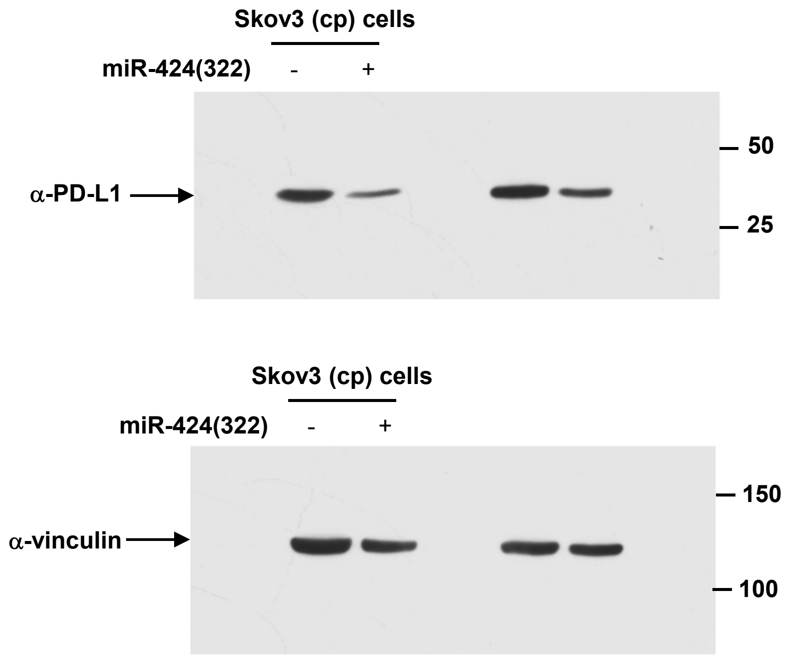

Supplementary Figure 9. Original blots for western blots in Figure 3E. miR-424(322) overexpression in Skov3 (cp) cells decreased the protein levels of PD-L1.

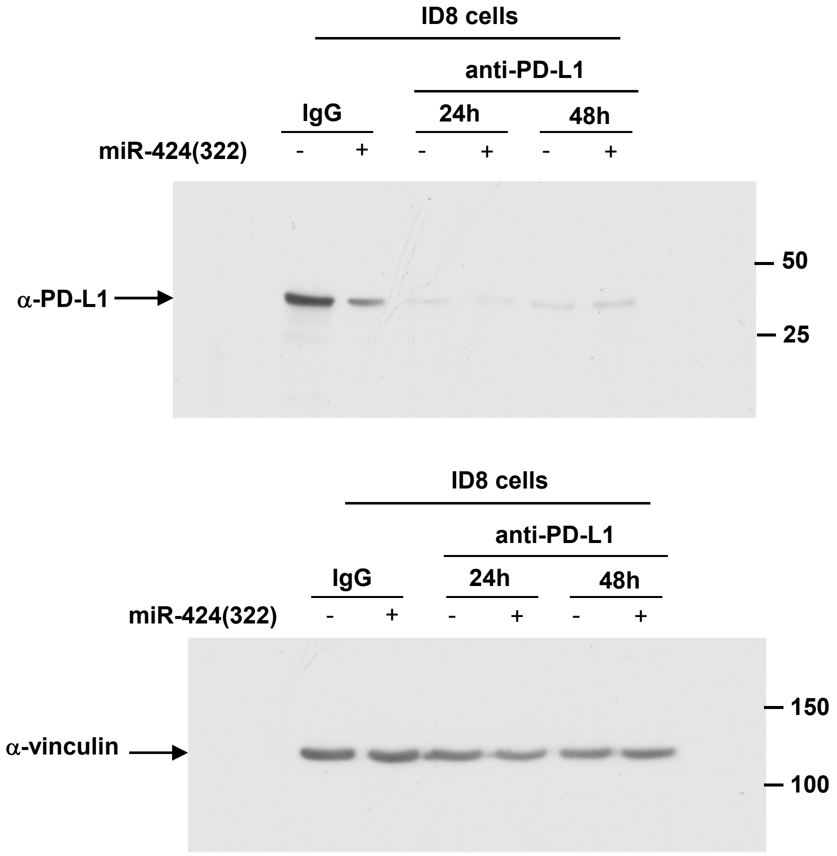

Supplementary Figure 10. Original blots for western blots in Figure 5B. miR-424(322) overexpression in ID8 cells decreased the protein levels of PD-L1.

Supplementary Table 1. Clinical characteristics of ovarian cancer patients.

Patient Characteristics (N = 42)		
**Characteristic**	**PFS > 6 (N = 21)**	**PFS < 6 (N = 21)**
Age		
< 50	5	4
> 50	16	17
Stage		
I-II	6	3
III-Ⅳ	15	18
Grade		
0	0	0
1	1	0
2	5	5
3	15	16
Histologic subtypes		
High-grade Serous	13	17
Low-grade Serous	2	2
Mucinous	3	1
Endometrioid	3	1
Debulking status		
Optimal (≤1cm)	8	7
Suboptimal (>1cm)	13	14
Chemotherapy response		
Sensitive	21	0

Resistant	0	21
